# Editorial: Genome Wide Association Studies and Genomic Selection for Crop Improvement in the Era of Big Data

**DOI:** 10.3389/fgene.2022.873060

**Published:** 2022-05-20

**Authors:** Alison R. Bentley, Charles Chen, Nunzio D’Agostino

**Affiliations:** ^1^ International Wheat and Maize Improvement Center (CIMMYT), El Batan, Mexico; ^2^ NIAB, Cambridge, United Kingdom; ^3^ Department of Biochemistry and Molecular Biology, 246 Noble Research Center, Oklahoma State University, Stillwater, OK, United States; ^4^ Department of Agricultural Sciences, University of Naples Federico II, Portici, Italy

**Keywords:** allele mining, genetic diversity, genotyping, high-throughput phenotyping (HTPP), genomic estimated breeding values (GEBV), genome-to-phenome

The exploitation of the genetic diversity of crops is essential for breeding purposes, as the identification of useful/beneficial alleles for target traits within plant genetic resources allows the development of new varieties capable of responding to the challenges of global agriculture (Food and Agriculture Organization of the United Nations, 2010).

Whole genome re-sequencing, genome skimming, fractional genome sequencing strategies, and high-density genotyping arrays enable large-scale assessment of genetic diversity for a wide range of species, including major and “orphan” crops (D’Agostino and Tripodi, 2017; Rasheed et al., 2017). This is however of limited value unless associated with adaptation and functional improvement of crops. Recently, several advances in high-throughput phenotyping have overcome the “phenotyping bottleneck” (Walter et al., 2015; Pieruschka and Schurr, 2019; Song et al., 2021), making available robust phenotypic data points acquired following the precise characterization of the agronomic and physiological attributes of crops. More and more studies are taking advantage of these scientific advances and of data science techniques to uncover the genome-to-phenome relationship and unlock the breeding potential of plant genetic resources. Genome-wide association studies (GWAS) and genomic selection (GS) are powerful data science approaches to investigate marker-trait associations (MTAs) for the basic understanding of simple and complex adaptive and functional traits (Liu and Yan, 2019; Voss-Fels et al., 2019; Varshney et al., 2021). Both approaches accelerate the rate of genetic gain in crops and reduce the breeding cycle in a cost-effective manner.

For this Research Topic we sought high-quality contributions, covering various aspects of genomics-assisted-breeding: increase in yield, improvement of nutritional content and end-use quality of crops, climate-smart agriculture, cropping systems in agriculture. We did not miss to ask for contributions on technical challenges related to the design of GWAS and GS experiments and data analysis.

Enhancing knowledge on (a)biotic stress tolerance of plants has a major impact on crop improvement strategies that aim to develop high yielding varieties in suboptimal environmental conditions.

Odilbekov et al. performed GWAS on a collection of nearly 200 winter wheat accessions to identify loci associated with seedling-stage resistance to *Septoria tritici* blotch (STB) disease, which is responsible for severe yield losses worldwide. Association tests with different statistical models returned a strong signal on chromosome 1B. Seven genes were identified as the most probable candidate genes for this QTL, as they play a key role in plant immunity and modulate the defense response. Finally, the authors demonstrated that the accuracy of the GS model for STB resistance can be improved when modeling GWAS associated variants as fixed effects.

Thapa et al. performed GWAS on a panel of 257 rice accessions to identify the QTLs and the underlying candidate genes responsible for cold tolerance and cold recovery during the germination phase. Their findings enrich the toolbox available to breeders for the development of new varieties with tolerance to low temperatures.

Hernández and Cortés subjected 78 geo-referenced wild common bean accessions to genotyping-by-sequencing (GBS) and derived three heat stress indices from phenotypic data points. Then, they applied the latest-generation GWAS models under a genome–environment association framework to identify putative loci underlying heat stress adaptation. The goal was to identify new sources of tolerance in the wild gene pool for use in breeding programs.

Increasing of crop yield potential is one of the main goals of breeding. Indeed, producing more with less is the key to feeding the growing world population. Within this motivating context, Zaïm et al., tested in the open field and in different environments four recombinant inbred line populations of durum wheat. GBS and the construction of a consensus linkage map led to the identification of over 30 QTLs for key agronomic traits. Six QTLs were found to be associated with grain yield and thousand kernel weight. The SNP markers anchored to these QTLs were then included as fixed effects into GS models, improving overall accuracy.

Sidhu et al. performed GWAS on a collection of almost 300 winter wheat accessions to determine SNP markers associated with coleoptile length. As a result, the authors identified eight candidate regions within which they found genes possibly involved in determining the target phenotype.

Many articles aimed to improve the predictive accuracy of GS models by considering some variables that influence traits or by proposing innovative technological solutions to fully exploit the genetic variability of plant genetic resources.

The article by Crossa et al. is about the comparison of the genome-based prediction accuracy of four methods: the additive genomic best linear unbiased predictor (GB), non-additive Gaussian kernel (GK), arc-cosine kernel (AK), and Deep Learning (DL). Single-environment and multi-environment G × E models on two real wheat datasets were used for benchmarking. Comparative analysis showed that AK outperformed the remaining methods, as it ensures competitive predictions at low costs in the tuning process.

Olatoye et al. identified main effect and epistatic effect loci of flowering time, maturity, and seed size in cowpea using a MAGIC population. Then, they used the identified quantitative trait nucleotides as fixed effects in parametric, semi-parametric, and non-parametric GS models and demonstrated that *a priori* knowledge of the genetic architecture of a trait improves prediction accuracy.

Allier et al. proposed adjustments to two parameters, namely the expected genetic value in the progeny (V) under a certain constraint on inbreeding (D), of the cross-selection strategy they published earlier. This arises from the need to consider within-family selection in recurrent genomic selection programs. The authors compared their UCPC-based optimal cross-selection strategy with the existing ones and proved that it was more efficient for converting genetic diversity into short- and long-term genetic gains.

Klápště et al. improved genomic predictions for traits with relatively low heritability and poor prediction accuracy by implementing multi-trait models based on the use of a marker-based relationship matrix, instead of classic pedigrees. The models applied to the diameter at breast height (target trait) did not outperform the multivariate model using all genetic markers in the case of the *Pinus radiata* population; conversely the strategy was advantageous in the case of the *Eucalyptus nitens* population, where the target trait had a low/moderate correlation with other heritable traits.

The untapped genetic variation preserved in germplasm banks serves as a source for future food and nutritional security for the globe. However, a major obstacle that prevents the use of bank accessions is the lack of adequate characterization and performance evaluation. In Kehel et al., 789 bread wheat landraces held in-trust at the gene bank of the International Center for Agricultural Research in the Dry Areas were scanned for seed traits and genetically evaluated using 12k DArTSeq SNP markers. Based on cross-validation, predicting untyped seed traits can be as accurate as 74% for seed width. Moreover, when incorporating climatic and environmental variables based on passport data, the prediction accuracy improved by an additional 8%. These findings advocate the advancement in predictive analytics and genomic technologies for identifying potential donors of desirable alleles for genetic introgression.

Considering the long reaction time and the expensive cost in conservation and sustainability of forest resources, Lstibůrek et al. conducted a multi-trait and multi-site large-scale genetic analysis with 4,625 25–35 years old European larch trees grown over 21 reforestation sites across four distinct climatic regions. In this study, the capacity of the marker-based pedigree information was demonstrated by comparing *in situ* heritability estimates. Furthermore, using this approach, a higher genetic response of the selected individuals can be expected for fitness and productivity attributes, suggesting that broad-spectrum climatic genetic evaluation can be an effective guiding principle for reforestation and genetic resource management without the reliance on structured tree breeding methods.

Finally, the last series of articles describes some data resources available for future studies or technical challenges related to the design of GWAS experiments.

Piot et al. analyzed over 1,000 *Populus trichocarpa* genomes to assess genomic diversity and identify rare and common alleles with high confidence for subsequent use in GWAS. Approximately 5% of the variants identified were non-synonymous and could represent rare defective genetic variants hypothetically associated with poplar phenotypic plasticity.

The mini-review by Srivastava et al. is quite different in content, as it provides an overview of the latest development of genetic and genomic resources in pearl millet and their use in GWAS and in the development of GS models for the estimation of GBEVs (genomic estimated breeding values).

The review by Pavan et al. provides advice on how to plan the experiments and choose the most appropriate and cost-effective genotyping method for crop GWAS. It also describes which quality control procedures should be applied on genotypic data points to avoid bias and false signals in genotype-phenotype association tests.

As genomics-driven knowledge advances rapidly and data science techniques for omics data continue to evolve and improve, the combination of the huge amount of genetic and phenotypic data points becomes more and more reachable. We looked at innovative examples whose purpose was to describe the genome-to-phenome connection and causation and to highlight the strengths and weaknesses of popular data mining strategies. We hope that the articles in this Research Topic can give further impetus to this area of research and can help expand the tools available to breeders.
